# Genomic surveillance and serological profile of SARS-CoV-2 variants circulating in Macaé and nearby cities, southeastern Brazil

**DOI:** 10.3389/fmicb.2024.1386271

**Published:** 2024-04-30

**Authors:** Amanda Cristina Veiga Fernandes da Silva, Carina Azevedo Oliveira Silva, Graziele Fonseca de Sousa, Viktoria Aparecida Gomes Silva Coelho, Lucas Tavares da Cunha, Artur Nunes Paes, Allan Pierre Bonetti Pozzobon, Daniele das Graças dos Santos, Raphael Mello Carpes, Evenilton Pessoa Costa, Cintia Monteiro-de-Barros, José Luciano Nepomuceno-Silva, Raquel de Souza Gestinari, Flávia Borges Mury

**Affiliations:** Laboratório Integrado de Doenças Emergentes e Negligenciadas, Instituto de Biodiversidade e Sustentabilidade (NUPEM) - Universidade Federal do Rio de Janeiro (UFRJ), Macaé, Brazil

**Keywords:** COVID-19, genomic surveillance, SARS-CoV-2, serology, variants

## Abstract

**Introduction:**

A characteristic of the COVID-19 pandemic has been the sequential emergence and global dissemination of SARS-CoV-2 variants, noted for their enhanced transmission efficiency. These variants with mutations in the Spike glycoprotein (S-glycoprotein), which interacts with ACE2 receptors in human cells is critical for infection, affects the transmissibility of the virus, which is a matter of great concern for public health.

**Objective:**

This research analyses the effects these variants on a cohort of vaccinated and naturally infected individuals from the cities of Macaé-RJ, Rio das Ostras-RJ, and Campos dos Goytacazes-RJ, Brazil, from March 2021 to March 2023.

**Methods:**

This investigation encompasses the Alpha (B.1.1.7), Gamma (P.1), Delta (B.1.617.2, B.1.671.3), and Omicron (BQ.1, BQ.1.1 sublines, and BF.7) variants, focusing on their genomic surveillance and implications for the disease’s epidemiology. The experimental analysis included a control group (vaccinated and uninfected subjects), and an infected group (post-vaccinated subjects). Samples from nasopharyngeal swabs underwent viral detection via RT-qPCR for diagnosis confirmation. RNase H-dependent RT-qPCR (rhAmp-PCR) and third-generation sequencing were used to detect SARS-CoV-2 variants. Anti-S-glycoprotein immunoglobulins were also evaluated for vaccinated infected and noninfected volunteers. Symptoms from infected individuals were compiled in order to reveal patterns of clinical signs associated with viral infection.

**Results:**

The study included 289 participants, with infections identified by Gamma (*n* = 44), Delta (*n* = 189), and Omicron (*n* = 56) variants. The prevalent symptoms among the naturally infected participants were cough, fever, sore throat, headache, and runny nose. For Omicron, cognitive symptoms such as memory loss and concentration issues were reported. Interestingly, the infected vaccinated group had higher anti-S-glycoprotein IgM production (*n* = 28, 0.2833 ± 0.09768 OD) compared to the uninfected vaccinated group (*n* = 14, 0.1035 ± 0.03625 OD). Conversely, anti-S-glycoprotein IgG production was higher in the control group (*n* = 12, 1.770 ± 0.1393 OD) than in the infected vaccinated group (*n* = 26, 1.391 ± 0.1563 OD).

**Conclusion:**

This comprehensive study enables monitoring of predominant variants and their correlation with clinical cases, providing valuable insights for public health. Our research group continues to survey circulating variants, contributing to the global understanding of the pandemic.

## 1 Introduction

The clinical presentation of COVID-19 varies among individuals and can range from asymptomatic to acute respiratory distress syndrome and multiple organ failure. Besides respiratory symptoms, the disease can also cause fever, cough, dyspnea, viral pneumonia, and other severe manifestations, such as heart and renal failure, and even death (Huang et al., 2020; World Health Organization, 2023). Routine diagnosis of COVID-19 relies on the patient’s epidemiological history, clinical presentation, and laboratory or point-of-care tests for confirmation (Corman et al., 2020; Wan et al., 2020).

RNA viruses exploit a plethora of genetic variation mechanisms to ensure their propagation. Consequently, mutational events in viruses can occur through a variety of mechanisms, including point mutations, insertions, deletions, recombination, reassortment, and template switching. Phylogenetic comparisons with other coronavirus strains, as well as previously reported recombination events between coronavirus strains, suggest that SARS-CoV-2 has undergone complex recombination events during its evolution (Singh and Yi, 2021).

During the infection process, the virus will make copies of its RNA genome, and at this point, replication errors can lead to mutations and rise of new SARS-CoV-2 variants. Indeed, it is well stablished that the SARS-CoV2 genome has been undergoing substantial evolutionary changes and diversification as it has spread globally. Thus, the successive waves of COVID-19 are still occurring worldwide due to the emergence and spreading of new viral variants. Pan-genomic analysis studies of global isolates of SARS-CoV-2 have revealed numerous genomic regions with greater genetic variation and distinct mutation patterns (Korber et al., 2020; Kumar et al., 2020).

Pharmaceutical industries and research centers around the world have developed several vaccines using either the complete Spyke glycoprotein (which mediates virus internalization in host cells through interaction with ACE2 membrane receptors), its fragments or even tis mRNA to induce to induce immunity against SARS-CoV-2 infection (Heinz and Stiasny, 2021). It has been widely shown that SARS-CoV-2 variants present several mutations that enable them to spread in the face of increasing population immunity, while retaining or expanding their replication robustness. These mutations belong to a repertoire of recurrent mutations, most of which are in the Spike glycoprotein coding gene, generating several changes in the Spike glycoprotein (Mascola et al., 2021). This glycoprotein is composed of two subunits, S1 and S2. In the S1 subunit, there is an N-terminal domain (NTD) and a receptor-binding domain (RBD). While subunit S2 comprises the fusion peptide (FP), heptapeptide repeat sequences 1 (HR1) and 2 (HR2), transmembrane domain (TM), and cytoplasmic domain (Huang et al., 2020; Yurkovetskiy et al., 2020; Suzuki and Gychka, 2021).

At the end of 2020, the WHO recommended categorizing new SARS-CoV-2 strains as either variants of interest (VOIs) or variants of concern (VOCs) (World Health Organization, 2023). VOIs are caused by mutations that lead to altered receptor binding, decreased antibody neutralization, weaker treatment effectiveness, increased disease severity, or enhanced transmissibility (CDC, 2021). VOCs are characterized by their transmissibility, disease severity, ability to evade humoral immunity, and decreased efficacy of treatments and vaccines (CDC, 2021; Tao et al., 2021). Thus, conducting genomic surveillance for SARS-CoV-2 is critical to ascertaining whether vaccines retain their effectiveness against prevailing variants.

Prior research has highlighted that the emergence of novel and evolving SARS-CoV-2 variants has necessitated a shift towards more adaptive diagnostic methods for detecting SARS-CoV-2 infections (Fernandes et al., 2022). It is essential to emphasize that SARS-CoV-2 can infect anyone, regardless of age or sex, ultimately leading to disease progression and potential death (WHO, 2020). The immune response plays a crucial role in the progression of COVID-19. While many individuals infected with SARS-CoV-2 remain asymptomatic or only develop mild symptoms (Mortaz et al., 2020), this virus can cause severe illness and even death in vulnerable groups.

This work aimed to track the occurrence and prevalence of SARS-CoV-2 strains in Macaé, a coastal municipality in southeastern Brazil, and its surrounding region, as well as to identify the primary symptoms associated with each circulating variant, and to evaluate the humoral response against them. Analyzing its factors in diverse populations can aid in our understanding of disease severity in susceptible individuals.

## 2 Materials and methods

### 2.1 Patient screening and exclusion criteria

**Figure 1:**
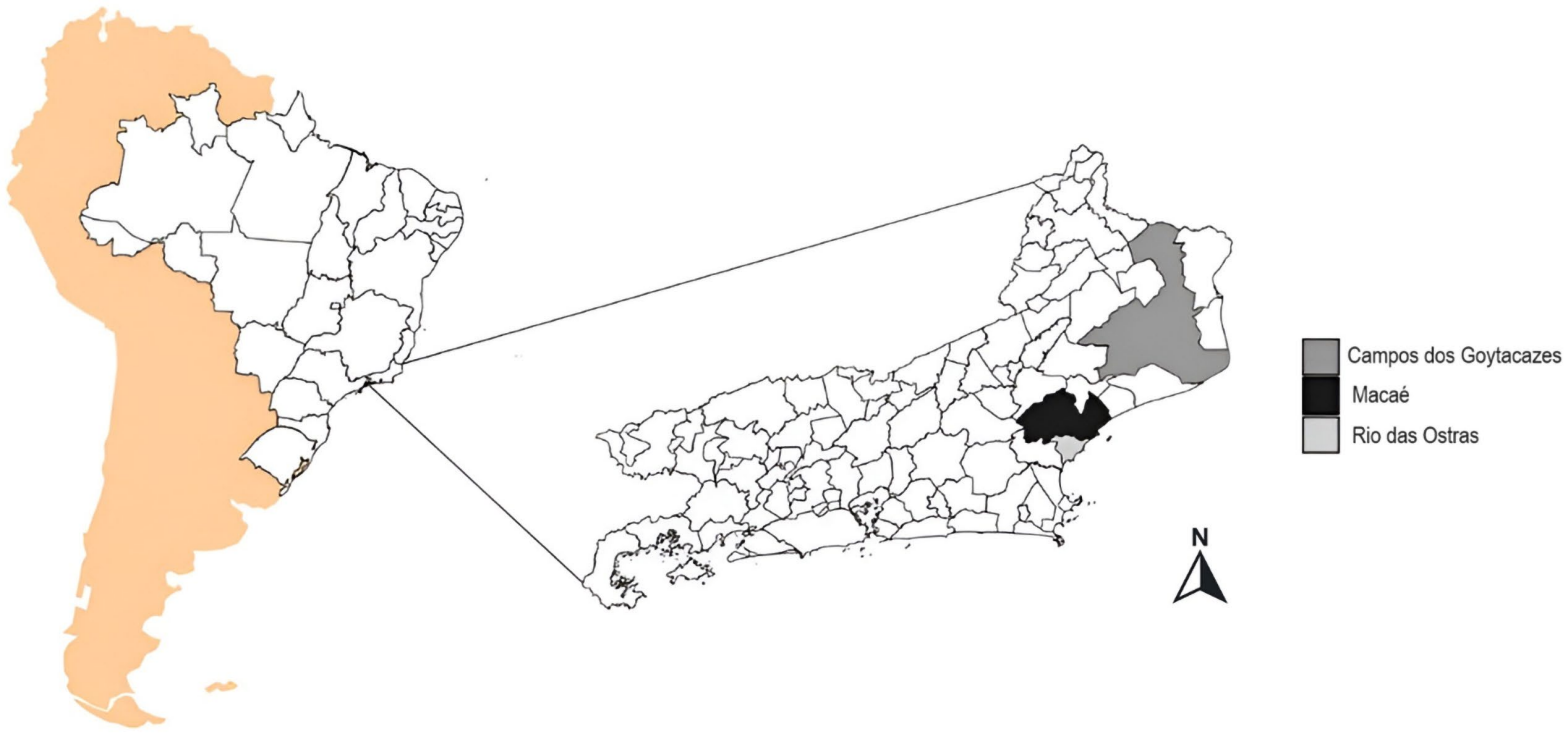
Location of the study area: Political map of the state of Rio de Janeiro, Brazil, with Macaé highlighted in blue, Campos dos Goytacazes in red, and Rio das Ostras in green. These cities were included in the genomic surveillance of COVID-19 from March 2021 to March 2023. Were used South America background from rnaturalearth1 and SF2,3 from the packages. Brazil map from geobr4. Rio de Janeiro map packages from IBGE, shapefile available at https://geoftp.ibge.gov.br/organizacao_do_territorio/malhas_territorais/malhas_municipais/municipio_2022/UFs/RJ/RJ_Municipios_2022.zip. Figure was generated by the authors using R 4.3.1 software version 1.1.5 (Team, 2023) and the ggplot2 Wickham (2016), ggspatial5, gridExtra (Auguie, 2017), and RColorBrewer packages available at (Neuwirth, 2014).

Patients who sought basic health care centers in the city of Macaé, and in two nearby cities (Campos dos Goytacazes and Rio das Ostras), located in the North Fluminense region the state of Rio de Janeiro, southeast of Brazil, were invited to participate in the study (Figure 1). The patients received information about the protocols of the research, and only those who agreed to the Term of Free and Clear Consent (TFCC) were included in the present study. The exclusion criteria were age below seventeen years old, pregnancy, or the absence of informed consent. This study was approved by the Research Ethics Committee of the National Health Council (protocol number: CAAE 57373422.8.0000.5699).

### 2.2 Test groups for serological analysis

Patient samples were collected between March 2021 and March 2023. Individuals presenting with flu-like symptoms who sought care within the Macaé Public Health System were initially screened using the rapid COVID-19 Ag test (Institute of Molecular Biology, Paraná, Brazil, batch: 220562Z115). Those who tested positive in the antigen test were selected for peripheral blood sampling via venipuncture for serologic analysis. Swab and blood samples from Campos dos Goytacazes and Rio das Ostras were provided by the Municipal Health Secretary. These patients completed a questionnaire to evaluate their signs, symptoms, comorbidities, sex, age, and vaccination status. A control group was composed of volunteers who had been vaccinated against COVID-19 and had not been infected with SARS-CoV-2.

### 2.3 Neutralizing antibody analysis

The S-UFRJ ELISA, as described by Alvim et al. (2022), was performed with the trimeric SARS-COV-2 Spike glycoprotein, kindly provided by the Laboratory of Cell Culture Engineering (LECC) from the Federal University of Rio de Janeiro (UFRJ). The protocols for semi-quantitative analysis of IgA, IgM, and IgG were previously described by Sousa et al. (2023). This analysis was performed using a Multiscan Sky microplate reader (Thermo Scientific) at 450 nm and results were expressed as the optical density (OD) or OD/cutoff ratio of the sample.

To quantify neutralizing antibodies, the Lumit^™^ SARS-CoV 2 Spike RBD: hACE2 Immunoassay Kit (Promega) was used, following the manufacturer’s recommendations. Luminescence was measured using the Fluoroskan FL microplate luminometer (Thermo Scientific) and results were expressed as relative light units (RLU). Following the manufacturer’s instructions, we included several pre-pandemic negative COVID-19 control samples for reference light output. These samples consisted of specimens collected at the Blood Bank of the Municipal Hemotherapy Service in January 2020, before the first case of COVID-19 was reported in Brazil.

### 2.4 Nasopharyngeal swab collection

To perform variant analysis the nasopharyngeal swab samples were collected, immersed in 2 mL of Dulbecco’s Modified Eagle Medium (DMEM—Gibco—11054020) and identified. Subsequently, the medium containing the sample were transferred to the cryogenic tubes and stored at −80°C until RNA extraction (Feitosa et al., 2021). All patients undergo a pre-screening process using a COVID-19 antigen test, with the exception of patients from the North Fluminense region, where screening is conducted in hospital units. Only samples that tested positive for COVID-19 are forwarded for confirmation via RT-qPCR. The 1,001 samples represent 995 patients tested at various times (refer to Supplementary Table S1). A subset of 54 patients underwent testing more than once, either within the same month counted as a single patient or across different months, in which case the test count was normalized.

### 2.5 Blood sample processing and storage

Blood samples were obtained via peripheral venipuncture, collected into 4 mL EDTA K3 tubes (Firstlab). Plasma was subsequently isolated through centrifugation at 3000 g using a Daiki DT-5000 for 10 min and preserved at −20°C for future analysis (Sousa et al., 2023).

### 2.6 Viral RNA extraction and RT-qPCR

Magnetic beads (Magmax Magnetic Kit, ThermoFisher) were used to extract RNA from swab samples according to the manufacturer’s instructions. RT-qPCR reactions for the confirmation of positive samples for SARS-CoV-2 were performed using the TaqMan^™^ reagent, as previously described in the Berlin (Corman et al., 2020; Feitosa et al., 2021) or CDC (2021) protocols. Samples were considered positive in RT-qPCR tests when two regions of the SARS-CoV-2 genome were amplified. Patients were considered uninfected when only the human internal control (RNAse P) was amplified. All RT-qPCR assays were performed on a QuantStudio 3 equipment (ThermoFisher Technologies, Waltham, Massachusetts, United States). Positive samples were also analyzed by the RNAse H-dependent PCR (rhAmp-PCR), to identify the SARS-CoV-2 variant (Beltz et al., 2018).

### 2.7 rhAmp-PCR

To identify the variants of SARS-CoV-2, rhAmp-PCR was performed (Beltz et al., 2018). This technique relies on a RNAse H2-dependent PCR (rhPCR) and allows the identification of some variants without the need for genomic sequencing. The cDNA was generated for amplification of fragments of the viral genome using specific kits (TaqPath 1-step multiplex master Mix/TaqMan^™^ SARS-CoV-2 mutation assays, ThermoFisher) by RT-qPCR in thermocyclers. For this, a total volume of reaction Mix was prepared with 2.5 μL TaqPath^™^ 1-step RTqPCR master Mix, CG (4×) (ThermoFisher); 0.25 μL TaqMan^™^ SARS-CoV-2 mutation panel assay (40×) (ThermoFisher); 4.75 μL of nuclease-free water and 2.5 μL of RNA. The following thermal protocol was performed for PCR amplification: Pre-reading at 60°C for 30 s; 10 Min at 50°C for reverse transcription; 2 Min at 95°C for rapid DNA polymerase activation; 45 cycles of 3 s at 95°C denaturation and 30 s at 60°C annealing and extension; and a post-read cycle at 60°C for 30 s. To distinguish between viral variants, FAM Dye reporters detected the mutant alleles, while the VIC dye reporter detected the reference allele. Data analysis was performed in QuantStudio^™^ design and analysis software v.2.5. Table 1 shows the representative SARS-CoV-2 mutations studied in this work. In some cases, where rhAmp-PCR was not sufficient, the viral genome was analyzed with the NGS technique, using the shotgun metagenomics approach. Oligonucleotides were purchased from ThermoFisher technologies (Waltham, Massachusetts, United States).

**Table 1:** SARS-CoV-2 mutations associated variant and lineages of origin.

Mutation	WHO label	Top associated variants and subvariants	Earliest documented samples	Assay ID
S.N460K.AAT.AAA	Omicron	BQ subvariants	Various countries	CV47VR3
ORF1ab.C7528T	Omicron	BF.7	Various countries	CV7DPCZ
ORF1b.Y264H	Omicron	BQ.1, BQ.1.1	Various countries	CV7DPCR
S339D + Q439R	Omicron	–	Various countries	CV47VRX + CVH49P2
S.delH69V70 + S.P681H.CCT.CAT	Alpha	B.1.1.7	United Kingdom	AN9HXTM + ANCFHV6
S.T20N.ACC.AAC + S.V1176F.GTT.TTT	Gamma	P.1	Brazil	CVCE3VA + CVKA3AU
S.L452R.CTG.CGG + S.T478K.ACA.AAA	Delta	B.1.617.2, B.1.671.3	India	CVAAAAD + CVNKRFP

The assay ID for each of these mutations was taken from the TaqMan SARS-CoV-2 mutation panel* (ThermoFisher). *https://www.thermofisher.com/br/pt/home/clinical/clinical-genomics/pathogen-detection-solutions/real-time-pcr-research-solutions-sars-cov-2/mutation-panel.html.

### 2.8 Next-generation sequencing and data analysis

Total RNA from SARS-CoV-2 positive samples was converted to cDNA using the SuperScript IV First-Strand Synthesis System (Thermo Fisher Scientific, United States). Viral whole-genome amplification was performed according to the Artic Network protocol^1^, using the SARS-CoV-2 primer scheme (V3). Sequencing libraries were constructed with the TruSeq DNA Nano kit (Illumina, United States) as described by the manufacturer. Libraries were sequenced in a MiSeq System with MiSeq Reagent Kit v3 (Illumina, United States) set to obtain 2 × 250 bp reads. Next, raw read sequences in FASTQ format were first pre-processed using FastQC (v0.11.4)^2^ and trimmomatic v0.39 (Bolger et al., 2014) for quality control and low-quality reads filtration, keeping those with an average quality ≥25. Bioinformatic pipeline for next-generation sequencing (NGS) data analysis includes removing optical duplicates with cutadapt v2.1 (Martin, 2011) and clumpify v38.41^3^, read mapping to the reference genome (NC_045512.2) using the BWA 0.7.17 (Li and Durbin, 2009; Martin, 2011), and post-processing steps with Samtools v1.10 (Li et al., 2009) and Picard v2.17.0 packages^4^. The sequence presented in the study is deposited in the GISAID (Khare et al., 2021), under accession number EPI_ISL_19049633.

### 2.9 Statistical analysis

Data analysis was performed with GraphPad Prism 8 software (GraphPad Software Inc., United States) and graphs were expressed as frequencies and percentages. Serological data were subjected to the Kolmogorov–Smirnov normality test. Data presenting normal distribution were submitted to unpaired, two-tailed Student’s *t*-test, while Mann–Whitney test was performed for non-parametric data. Results were considered significant whenever *p* < 0.05 (95% confidence level).

To evaluate the dissimilarities between the vaccinated groups based on the frequency of symptoms and SARS-CoV-2 variants, an analysis of principal components (PCoA) was performed in past software version 4.03 and Jaccard similarity index.

## 3 Results

### 3.1 SARS-CoV-2 circulating variants

**Figure 2:**
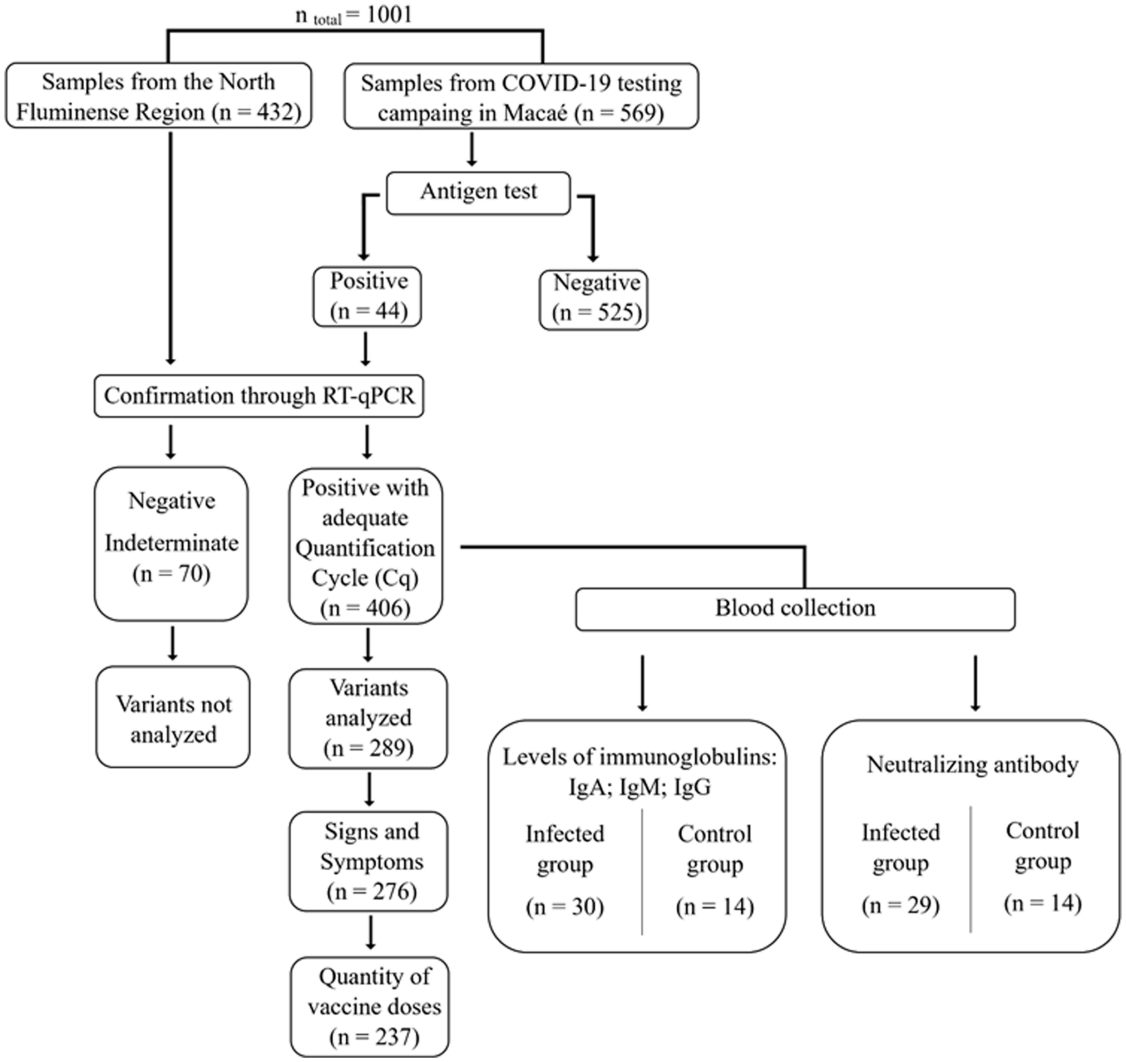
Flowchart of the experimental procedure and positive samples used in genomic surveillance and serological analysis.

During the study period, a total of 1,001 volunteers were able to participate and had their samples collected. Of these, 476 subjects tested positive for SARS-CoV-2 with the rapid antigen test and were subjected to RT-qPCR to confirm positivity. 289 samples were further analyzed to identify variants, and 30 of these samples were submitted to the semi-quantitative S-UFRJ ELISA antibody test (Figure 2).

**Figure 3:**
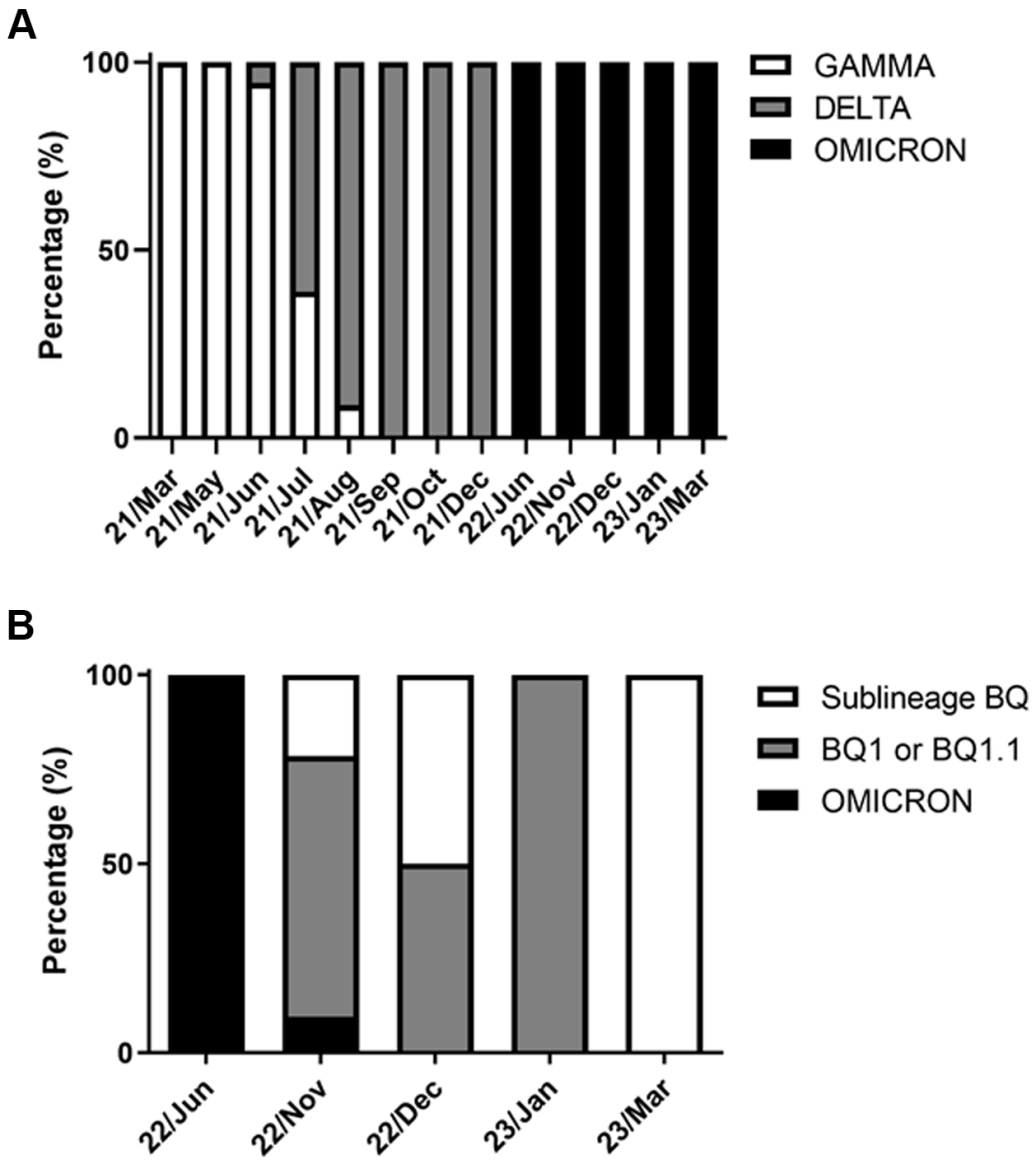
SARS-CoV-2 variants circulated in the cities of Macaé, Rio das Ostras, and Campos dos Goytacazes from March 2021 to March 2023. The data are expressed as a percentage (%) of the total cases analyzed. **(A)** Variants (GAMMA, *n* = 44; DELTA, *n* = 189; and OMICRON, *n* = 56); and **(B)** analysis of the OMICRON subvariants (omicron, *n* = 9; BQ1 or BQ1.1, *n* = 33; and BQ, *n* = 14). Notably, the GAMMA and DELTA variants co-occurred between June and August 2021, with the Omicron variant predominating from June 2022 onwards. The variants were identified using the rhAmp-PCR method or genome sequencing.

The rhAmp-PCR analysis for SARS-CoV-2 variants revealed that 44 samples were positive for the Gamma variant, 189 were positive for the Delta variant, and 56 for the Omicron variant (Figure 3A). From March 2021 to August 2023, almost all samples were positive for the Gamma variant. In contrast, the Delta co-occurred with Gamma variants from June 2021 to August 2021 and Gamma variant predominated from September to December 2021. The Omicron variant was introduced in the northeastern region of Rio de Janeiro Estate by June 2022 and remained present until March 2023. In December 2022, Omicron sub-variants started to predominate in the samples in which BQ and BQ.1 were identified (Figure 3B).

### 3.2 Signs and symptoms of COVID-19 infection

**Figure 4:**
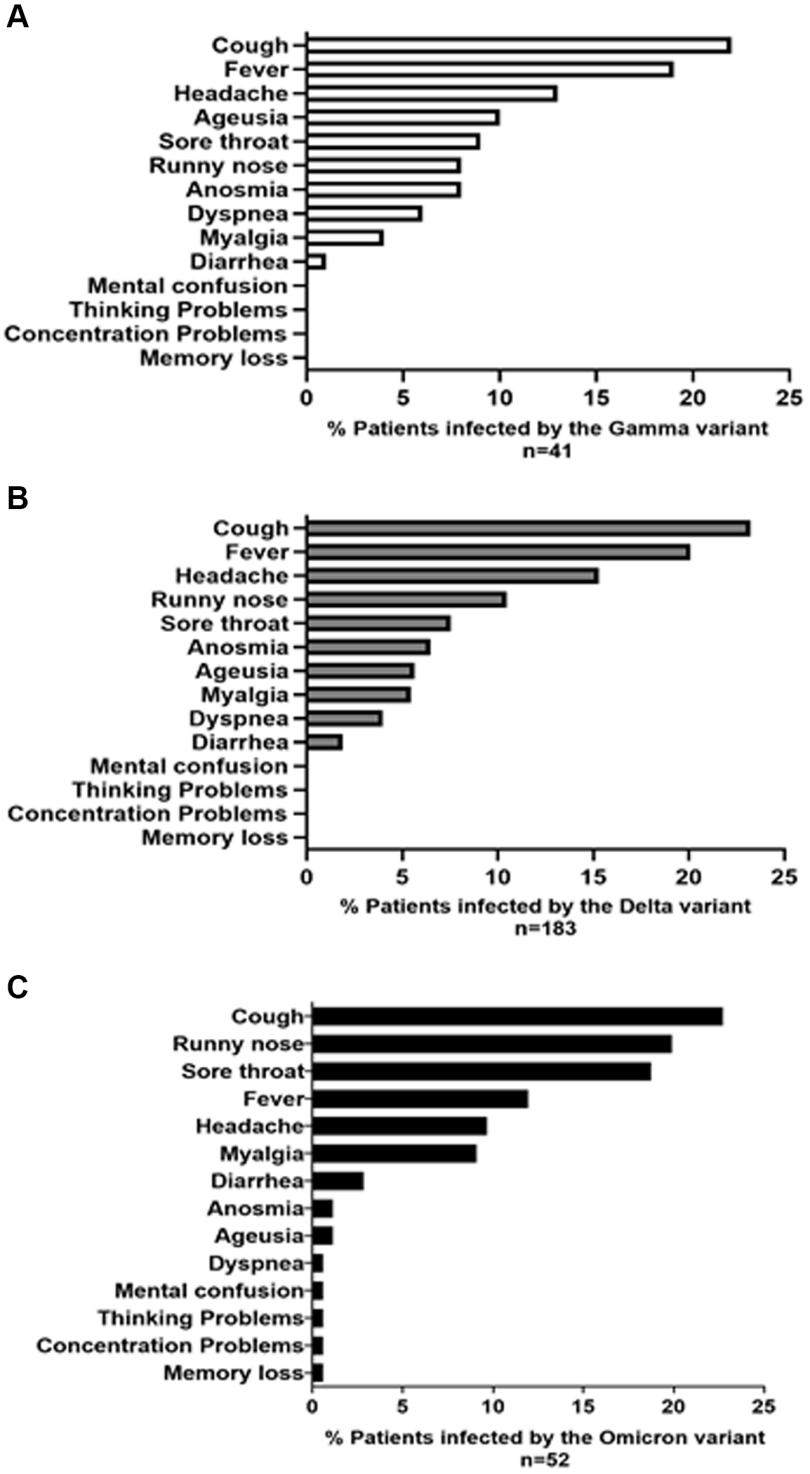
The prevalence (%) of symptoms and signs reported by patients infected with the **(A)** GAMMA **(B)** DELTA, and **(C)** OMICRON variants is presented. The symptom profile and frequency were reported by 276 volunteers, including 41 infected with the GAMMA variant, 183 with the DELTA variant, and 52 with the OMICRON variant, spanning from March 2021 to March 2023.

Figure 4 illustrates 14 common signs and symptoms observed among patients infected with Gamma (Figure 4A), Delta (Figure 4B) and Omicron (Figure 4C) variants. The most frequent symptoms for Gamma and Delta variants were cough, fever, and headache (Gamma: 22, 19, and 13; Delta: 111, 96, and 73). Among the 52 patients infected with the Omicron variant, there were a total of 176 reported signs and symptoms. The most prevalent symptoms were cough, accounting for 40 out of 176 reports (22.7%), runny nose with 35 reports (19.9%), and sore throat with 33 reports (18.7%). Furthermore, a small subset of individuals diagnosed with the Omicron variant exhibited symptoms such as mental confusion, attention deficit, thought disorder, and memory loss (Figure 4C). Notably, individuals infected by the Gamma and Delta variants did not report neuronal symptoms, as observed for the Omicron.

### 3.3 Vaccine versus variants

**Figure 5:**
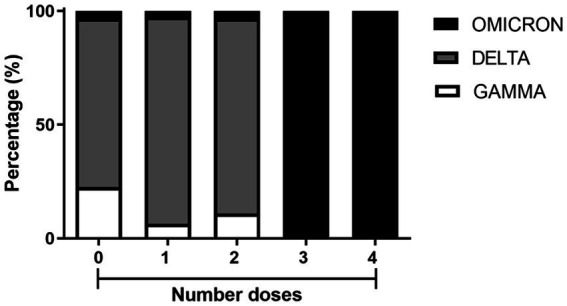
Percentage of SARS-CoV-2 variant occurrences in residents from Macaé, Rio das Ostras, and Campos dos Goytacazes who received one, two, three, and four doses of COVID-19 vaccines, compared with unvaccinated residents. Among the unvaccinated, there were 7 cases of the GAMMA variant, 23 of the DELTA variant, and 1 of the OMICRON variant. Among those who received one dose, there were 5 cases of GAMMA, 71 of DELTA, and 2 of OMICRON. For those who received two doses, there were 10 cases of GAMMA, 79 of DELTA, and 3 of OMICRON. Among those who received three doses, there were 17 cases of OMICRON. Finally, among those who received four doses, there were 19 cases of OMICRON. These samples were collected from March 2021 to March 2023.

To estimate vaccine effectiveness against symptomatic disease caused by the Delta, Gamma and Omicron variants, a total of 237 patients were assessed for the vaccination status (Figure 5). As expected from the timeline of variant emergence and the local vaccination schedule, individuals who were either unvaccinated or had received one or two doses of the vaccine were primarily infected with the Delta and Gamma variants. Additionally, patients who received three or four doses of vaccination were infected with the Omicron variant (Figure 5). Among all patients, 201 out of 237 (84.8%) contracted the virus despite having received 0–2 doses of the vaccine. In contrast, only 36 out of 237 (15.2%) became infected after receiving 3–4 doses. These data suggest a 6.5-fold reduction in infections after the third vaccine dose.

Upon analyzing the types of vaccines administered to patients, we observed the following distribution: the first dose was predominantly AstraZeneca (99/198, 50%), followed by CoronaVac (50/198, 25%), Pfizer (44/198, 22%), and Janssen (5/198, 3%). For the second dose, the majority received AstraZeneca (67/123, 54%) and CoronaVac (39/123, 32%), with a smaller percentage receiving Pfizer (17/123, 14%). With the third dose, there was a significant increase in the administration of Pfizer (26/34, 76%), while the other vaccines represented a smaller percentage (AstraZeneca: 5/34, 15%; CoronaVac: 2/34, 6%; Janssen: 1/34, 3%). Lastly, for the fourth dose, there was a more balanced distribution between Pfizer (9/18, 50%) and AstraZeneca (6/18, 33%), with a smaller proportion receiving Janssen (3/18, 17%) (Supplementary Figure S1).

### 3.4 Comparison using principal coordinates analysis

**Figure 6:**
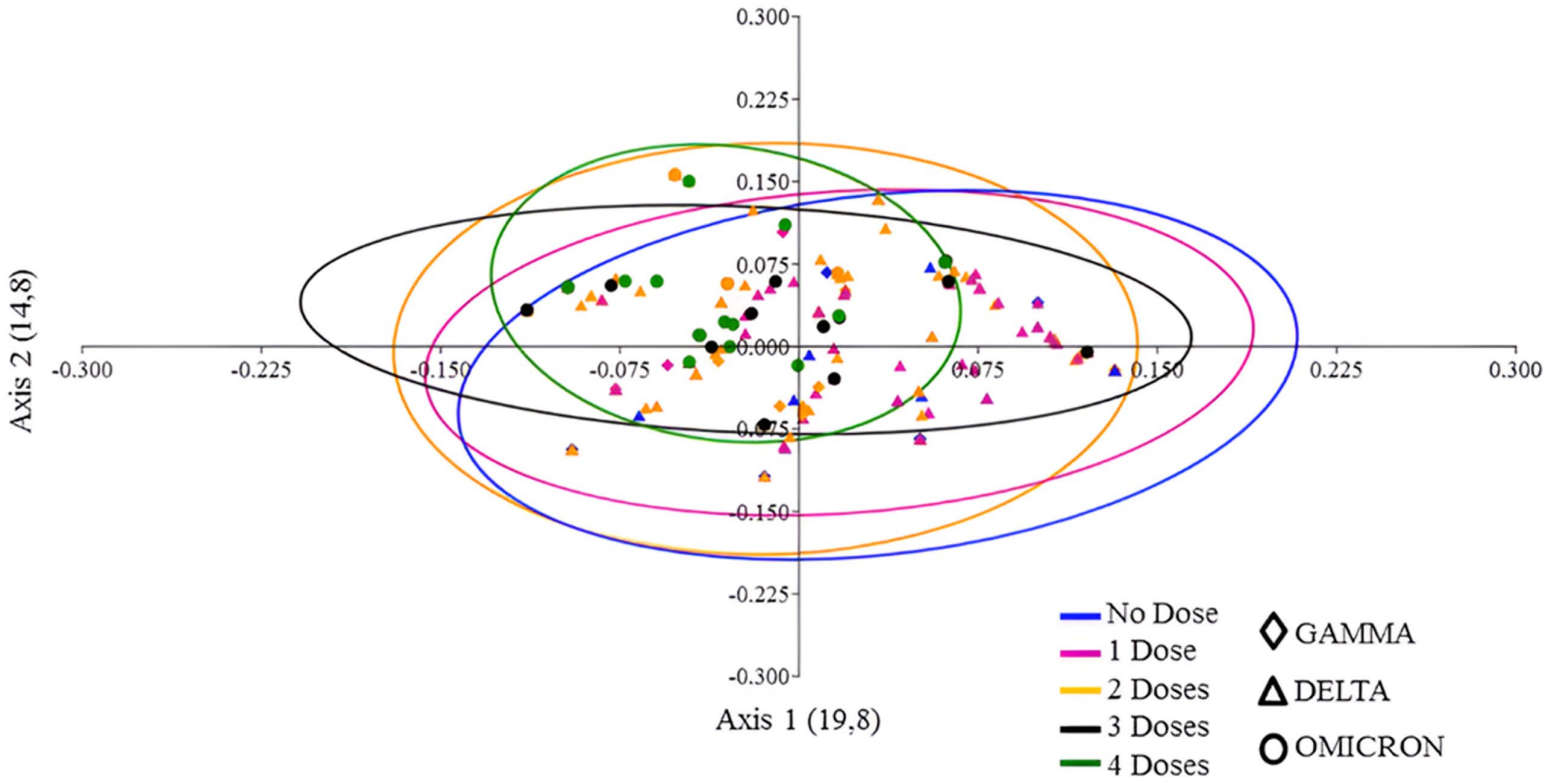
PCoA plot of vaccine doses and SARS-CoV-2 variants, utilizing presence/absence data on the frequency of signs and symptoms and a Jaccard distance matrix. The colors in the plot represent the number of vaccine doses, while the symbols denote the variants that circulated in the cities of Macaé, Rio das Ostras, and Campos dos Goytacazes between March 2021 and March 2023.

To analyze the congruence between vaccination status and clinical presentations of COVID-19 we measured similarity the extent of clustering similar topologies using principal coordinates analysis (PCoA). Figure 6 reveals that patients who received one or two doses were widely dispersed, that is, they presented a broad spectrum of clinical manifestations. However, the Gamma cloud (diamond) for one dose (pink) tended to cluster in a different region than patients who received two doses (yellow). A similar trend can be observed for the Delta variant (triangle) (Figure 6). Patients who received either three (black) or four (green) vaccine doses were infected with the Omicron variant (circle). The PCoA revealed that patients who received either three or four doses of the vaccine did not have significant differences. Nevertheless, they were closer together and formed a smaller cluster compared to patients who received up to two doses of the vaccine (Figure 6).

### 3.5 Immunoglobulin profile

**Figure 7:**
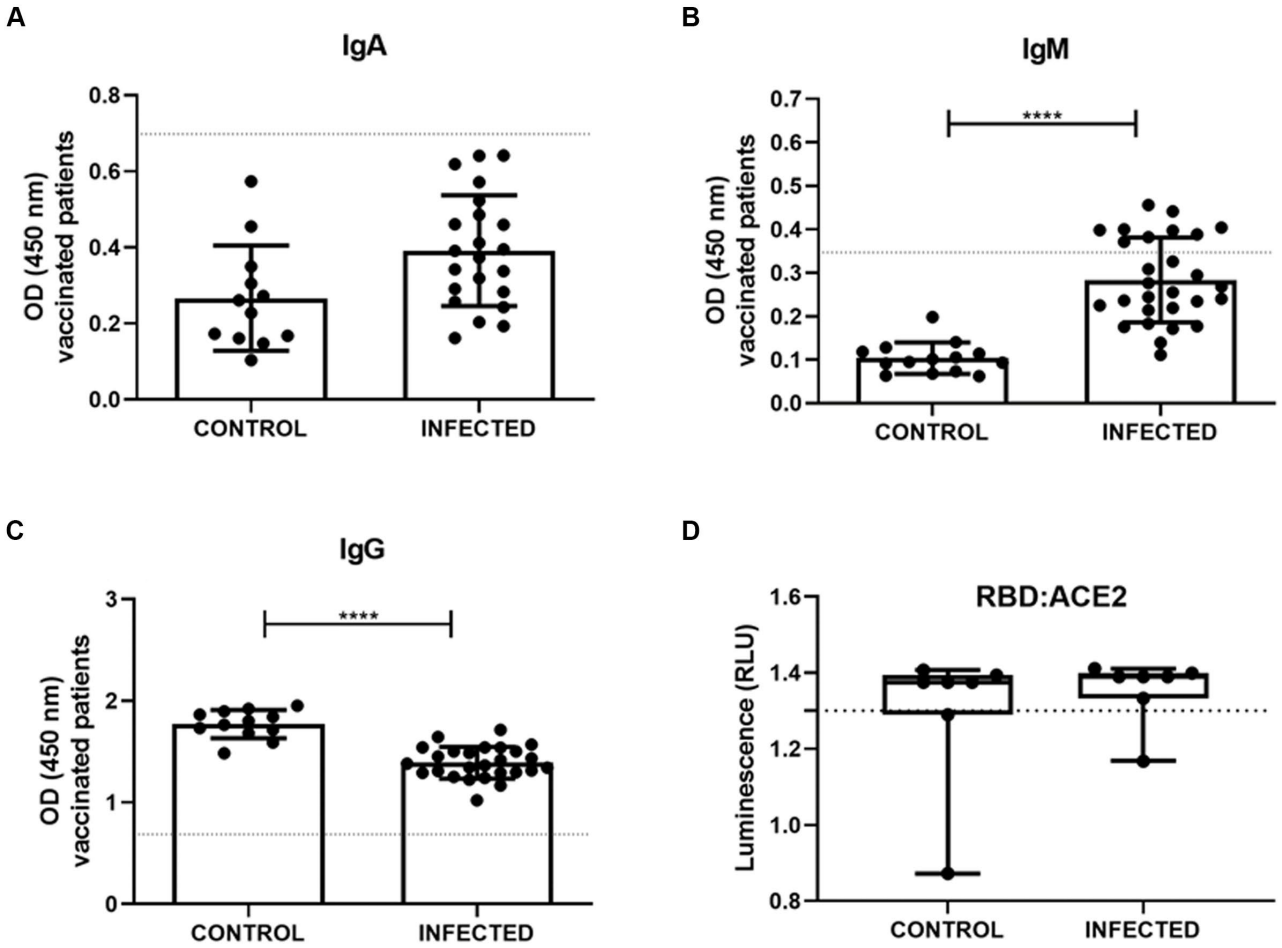
Serum measurements of IgA **(A)**, IgM **(B)**, IgG **(C)** were obtained through S-UFRJ-ELISA assays in a total of 42 patients, of whom 14 were vaccinated without COVID-19 (CONTROL; *n* = 12; 14 and 12; respectively) and 28 were vaccinated and naturally infected with the virus (INFECTED; *n* = 22; 28 and 26; respectively). **(D)** Lumit SARS-CoV-2 Spike RBD: hACE2 comparison of sensitivity of the samples from vaccinated volunteers without COVID-19 (CONTROL; *n* = 14) and infected vaccinated patients (INFECTED; *n* = 29). The dotted line denotes the cutoff value for positive levels. Normal distribution of data was assessed using the Kolmogorov–Smirnov test. Parametric data were statistically analyzed using the unpaired *t*-test **(A–C,E)**; non-parametric data using the Mann–Whitney test **(D)**. **** indicates *p* < 0.0001.

We further examined the profiles of anti-S-glycoprotein IgA, IgM, and IgG, from vaccinated patients who were either infected or not infected with SARS-CoV-2. The semiquantitative analysis of IgA showed that control and infected patients were negative for IgA, presenting optical density (OD) below the cut-off (Figure 7A). IgM analysis of the infected individuals showed an increase in the production of this antibody class (*n* = 28, 0.2833 ± 0.09768) compared to the control group (*n* = 14, 0.1035 ± 0.03625; Figure 7B). Notably, the IgG exhibited a significant difference between control and infected groups, in which infected subjects presented less IgG (*n* = 26, 1.391 ± 0.1563) when compared to the control uninfected ones (*n* = 12, 1.770 ± 0.1393; Figure 7C). The majority of the samples surpassed the cut-off value of 1.3, indicating effective interaction between the immunoglobulin and the S-glycoprotein receptor-binding domain (RBD), thereby demonstrating neutralizing capacity (Figure 7D). Infected patients exhibited antibody neutralization values distributed as follows: 25th percentile at 1.333, median at 1.39, and 75th percentile at 1.399. For non-infected patients, the values were 25th percentile at 1.29, median at 1.375, and 75th percentile at 1.394. Furthermore, there were no statistically significant differences in the neutralizing potential of immunoglobulins between infected and uninfected patients with SARS-CoV-2 (Figure 7D).

## 4 Discussion

The emergence of new SARS-CoV-2 variants is a continuous process of pathogen evolution that significantly increases uncertainty about the future. This study investigates the clinical profile caused by different SARS-CoV-2 variants, with the intention of to comprehend if the mutations that characterize each variant are able to enhance infection and/or immune escape. It also reports the frequency of recurrent signs and symptoms in vaccinated patients infected with SARS-CoV-2 variants during the period of COVID-19 vaccination ranging from March 2021 to March 2023.

The region under study is known for being the main hub of the Brazilian oil industry and related activities, attracting people from various parts of Brazil and the world. According to Castro et al. (2003), around twenty thousand individuals commute to and from Macaé daily. In the first 2 years of the COVID-19 pandemic, the municipality of Macaé implemented public policies that included social isolation strategies, health barriers, mass testing, and specific health units to care for people infected with SARS-CoV-2. As a result, the city has achieved one of the lowest mortality rates due to CVID-19 in Rio de Janeiro (Feitosa et al., 2021). This study also includes the municipality of Rio das Ostras, which shares urban borders with Macaé, with many residents and workers transiting daily between these two cities. The other municipality included is Campos dos Goytacazes, situated at about 100 km of Macaé and representing the largest and densely populated urban center in the north region of Rio de Janeiro State.

From March 2021 to March 2023, Macaé and nearby cities experienced the presence of the Gamma, Delta, and Omicron variants, which were also detected globally during that period (Hadj Hassine, 2022; Umair et al., 2022). Interestingly, there was a period, from December 2021 to November 2022, when very few positive samples were identified in this study (Figure 3A). Potential factors explaining this occurrence are heightened vaccination rates and reduced virus circulation in the area, resulting in a decreased demand for COVID-19 rapid tests. This gap may have been influenced by rapid tests with negative results and RT-qPCR with indeterminate results (Supplementary Figure S2).

From March 2021 to March 2023, Macaé and nearby cities experienced the presence of the Gamma, Delta, and Omicron variants, which were also detected globally during that period (Hadj Hassine, 2022; Umair et al., 2022). Interestingly, there was a period, from December 2021 to November 2022, when very few positive samples were identified in this study (Figure 3A). Potential factors explaining this occurrence are heightened vaccination rates and reduced virus circulation in the area, resulting in a decreased demand for COVID-19 rapid tests. This gap may have been influenced by rapid tests with negative results and RT-qPCR with indeterminate results (Supplementary Figure S2).

Genomic sequencing analysis has confirmed that the Omicron variant, when compared with the Alpha, Beta, Gamma, and Delta variants of concern, carries a significant number of mutations relative to the original SARS-CoV-2 genome isolated from Wuhan. Notably, several of these mutations are unusual or novel (Dhama et al., 2023). Specifically, the spike glycoprotein gene of the Omicron variant has at least 32 mutations, which is twice the number found in the Delta variant (Tian et al., 2021). This study utilized five mutant alleles to identify the Omicron variant, including two for the original lineage and three for the BQ subvariants (BQ.1, BQ.1.1, and BF.7) (Table 1). As shown in Figure 3B, the BQ subvariants in the study area were first detected between November and December 2022, and reemerged in March 2023.

It is well accepted that due to dynamic changes in the genome of SARS-CoV-2, its symptoms have also varied considerably (Torabi et al., 2023). This study analyzed data from patient populations who exhibited signs and symptoms indicative of COVID-19 upon health care center admission. Our results showed that patients infected with the Gamma, Delta, and Omicron variants reported cough, fever, headache, ageusia, sore throat, runny nose, anosmia, dyspnea, myalgia, and diarrhea. Studies conducted in diverse regions of the world have yielded similar results to ours when concerning the frequency of signs and symptoms (Bhattacharya et al., 2021; Saegerman et al., 2021; Bazargan et al., 2022). Monteón et al. (2023) observed that the Delta variants are associated with respiratory and digestive symptoms, whereas Omicron was more closely associated with articular and digestive symptoms; finally, the Gamma variant displayed wider and more diverse symptoms. Likewise, a small proportion of patients from Macaé and nearby cities infected with the Omicron variant reported experiencing four additional symptoms: memory loss, concentration problems, reasoning difficulties, and mental confusion. In our sampled patients, the Gamma and Delta variants were never associated with these symptoms. It is important to note that neuroinvasiveness, neurotropism, and neurovirulence appear to be consistent traits across various SARS-CoV-2 variants, albeit with varying degrees of severity. Actually, all SARS-CoV-2 variants demonstrate neuroinvasiveness, irrespective of the specific clinical manifestations they provoke (de Melo et al., 2023).

In 2020, several SARS-CoV-2 variants (including Alpha, Beta, Gamma, and Delta) appeared and spread worldwide, becoming prominent epidemic strains in many countries (Faria et al., 2021; Leung et al., 2021; Makoni, 2021; Singh and Yi, 2021, respectively). Compared with the early wild-type strain, they accumulated many mutations in the spike glycoprotein gene, which led to these variants of concern being more transmissible and even more capable to escape the adaptative immune response (Tian et al., 2021, 2022). For example, by June 2021, the Delta variant became dominant worldwide (CNBC, 2021) and reached the city of Macaé and its surroundings, becoming dominant in July 2021. At that time, the COVID-19 symptoms reported for Delta variant were very similar to those caused by infection with the Gamma variant (see Figures 4A,B). However, hospitalizations in Macaé due to COVID-19 decreased at that time (according to Macaé City Hall, 2021). Of the infected volunteers with available vaccination information, 63.29% were patients who had received up to two doses of the vaccine at the time of Delta variant circulation (Figure 5). The Omicron variant appeared latter in mid-2022 and mostly infected patients who had received up to four doses of the vaccine (Figure 5). As a consequence, the Omicron variant did not cause most of the health complications observed by occasion of pre-vaccination infections.

Several studies have shed light on how mutations in the SARS-CoV-2 spike protein gene influence viral neutralization by the immune system (Weisblum et al., 2020; Andreano et al., 2021). It has been noted that the variants of concern have accumulated a significant number of mutations in this gene, enhancing the virus’s transmissibility and ability to evade the immune response compared to the early wild-type strain (Harvey et al., 2021; Tian et al., 2021). Notably, mutations at the 477th position of the spike protein gene (S477G, S477N, and S477R) are among the key monoclonal antibody evasion mutations reported, with the S477G mutation conferring resistance to two out of four analyzed serums (Liu et al., 2021).

Vaccines have exerted a significant impact on various SARS-CoV-2 variants and associated symptoms. Initially developed to target the original strain of the virus, they have also demonstrated efficacy against newer variants, such as Omicron. Vaccination has played a pivotal role in mitigating the severity of symptoms, hospitalizations, and fatalities linked to COVID-19, especially among older adults, frontline workers, and individuals with underlying health conditions. The advent of new variants underscores the necessity for sustained vaccination efforts and ongoing research and surveillance of viral evolution. To maintain efficacy against emerging variants, vaccines may require periodic updates (McLean et al., 2022; Malik et al., 2022a, b).

The COVID-19 vaccination program commenced in Macaé and nearby cities when the Gamma variant was prevalent in the region. Data from Macaé City Hall revealed that the hospital bed occupancy rate decreased from 64% in March 2021 to 56% in July, after the local vaccination campaign began. According to the WHO report from 2023, the effectiveness of the vaccination campaign against the Gamma variant reduced its transmission and lethality (WHO, 2023). As the vaccination coverage increased in the following months, the number of cases and deaths gradually decreased until a new wave of cases emerged in January 2022.

Just like molecular testing, serologic testing is essential for epidemiologic surveillance of SARS-CoV-2 (Ong et al., 2021). These tests reveal the presence of anti-SARS-CoV-2 antibodies, including IgA, IgM, IgG, or total antibodies (Mekonnen et al., 2021; Ong et al., 2021). As occurs with other infectious diseases, in the early stages of a SARS-CoV-2 infection, specific IgM levels are typically high (Ong et al., 2021), which is also observed in the vaccinated/infected group in the study. Research has shown that IgA and IgM antibody levels decline rapidly following a SARS-CoV-2 infection, while IgG levels rise and remain steady for at least four to six months after the infection (Gudbjartsson et al., 2020; Isho et al., 2020; L’Huillier et al., 2021). According to recent studies, patients present detectable levels of IgG antibodies within seven to fourteen days of COVID-19 symptoms onset (Long et al., 2020; Ong et al., 2020). Conversely, our study detected significantly reduced levels of IgG in the vaccinated/infected group, which may me associated to a partial immune escape which is frequently observed for SARS-CoV-2. Our data suggest that the acquired defenses should be kept in high levels to reduce the impact of highly transmissible and immune-escaping variants like Omicron. Thus, booster vaccine shots are highly recommended. Recent data suggest that increased IgG levels are associated with a decreased risk of infection (Eberhardt et al., 2022).

The mucosal surfaces of the human body serve as the first line of defense against viral infection. In this regard, as SARS-CoV-2 is known to infect humans via the respiratory system, oral mucosa, and conjunctival epithelium, mucosal IgA plays an vital role in the formation of these physical and biochemical barriers (Paces et al., 2020; Rizzo et al., 2020). Prior research has shown that within the first week of infection, 75% of patients have a detectable IgA response, which appears to be stronger and more durable than the IgM response (Padoan et al., 2020; Rizzo et al., 2020). Similarly, in severe COVID-19 patients, Okba et al. (2020) observed a trend toward an increase in IgA response. Contrary to expectations, our analysis did not reveal any discernible difference in IgA levels during the early phase of infection.

Based in the correlation between anti-Spike IgG levels and neutralizing activity, some studies have suggested that protection against SARS-CoV-2 reinfection may last 1.5–2 years, with potentially longer-lasting defense against serious infections (Gaebler et al., 2021; Wei et al., 2021). However, the duration of protection may be shortened by the emergence of variants that require higher levels of antibodies for neutralization (Wei et al., 2021).

The emergence of the Omicron variant of SARS-CoV-2 has raised concerns due to its potential to evade neutralizing antibodies, significantly impacting both vaccinated and previously infected individuals. Studies indicate that over 85% of neutralizing antibodies are ineffective against the Omicron strain, including those from clinically approved antibody therapies (Cao et al., 2022; Cui et al., 2022; Wang et al., 2023). Surveys conducted in many countries have shown varying rates of reinfection among individuals previously infected with different variants of SARS-CoV-2. For instance, in Brazil, in the beginning of 2021 the northern region (with a seroprevalence of 29%) had higher incidences of reinfection compared to the southeast region (with a seroprevalence of 12%) (Figueiredo et al., 2023). The escalation in COVID-19 cases was predominantly ascribed to the advent of the Gamma variant (P.1) in the Amazon region. A comprehensive study conducted in the United Kingdom unveiled a spectrum of reinfection rates, demonstrating a progressive diminution in cross-immunity, especially with disparate variants (Office for National Statistics, 2020). A confluence of factors, including the inherent characteristics of the virus, vaccination status, and host variations, can profoundly influence the reinfection. As SARS-CoV-2 persists accumulating mutations, it becomes imperative to prioritize unceasing vaccination initiatives and personal protective measures (Mengist et al., 2021).

Genomic surveillance studies are crucial for monitoring the emergence of future coronavirus variants, also helping in predicting their ability to evade immunity (Vasireddy et al., 2021; Buisson, 2022). In this study, we mapped the variants that circulated in the municipality of Macaé and of two nearby cities, generating relevant data to support epidemiological control strategies. Our study, while contributing valuable insights, is limited by a relatively small sample size and a lack of specific vaccine analysis. To achieve greater precision, we must conduct large-scale studies with long-term follow-ups. Such studies will enable us to obtain more comprehensive epidemiological profiles and will also contribute to a better understanding of the impact of vaccines.

## 5 Conclusion

The efficacy of vaccines may be undermined by the swift emergence and proliferation of SARS-CoV-2 Variants of Concern (VOCs) that have the potential to evade neutralizing antibodies and/or cell-mediated immunity. Additionally, while rare, significant adverse events have been reported shortly after the administration of both viral vector and mRNA vaccines. Our research emphasizes the follow-up and evaluation of clinical severity, particularly among vaccinated individuals, in the face of the emergence of new variants of concern, such as Omicron, characterized by a high number of mutations. We have discerned symptomatic differences across variants, which can be regarded as a critical factor for precise diagnosis and treatment, especially considering that Omicron is associated with cognitive symptoms, such as memory loss. While vaccines have significantly mitigated severe complications, certain variants and subvariants may still infect individuals, even after receiving multiple doses. Thus, it is essential to consistently examine the effectiveness of vaccines in the fighting against new variants that may emerge. Variants like Omicron, capable of evading neutralizing antibodies, raise concerns about escalated reinfection rates, even among previously infected individuals. Therefore, it is crucial to prioritize ongoing vaccination campaigns, implement strict preventive measures, and consider the dynamic interplay between viral evolution, individual immunity, and the efficacy of existing treatments and vaccines. Our study emphasizes the need for continuous surveillance, variant monitoring, ongoing vaccine development, and the implementation of adaptive public health measures to combat the evolving nature of SARS-CoV-2. In conclusion, it is crucial to prioritize ongoing surveillance and variant monitoring in tandem with continuous vaccine development efforts. The implementation of adaptive public health measures, which take into account the interplay between viral evolution, individual immunity, and treatment efficacy, will be vital in effectively combating the dynamic nature of SARS-CoV-2.

## Data availability statement

The datasets presented in this study can be found in online repositories. The data presented in the study are deposited in the GenBank repository, accession number PP693357.

## Ethics statement

The studies involving humans were approved by Research Ethics Committee of the National Health Council (protocol number: CAAE 57373422.8.0000.5699). The studies were conducted in accordance with the local legislation and institutional requirements. The participants provided their written informed consent to participate in this study.

## Author contributions

AS: Conceptualization, Investigation, Methodology, Validation, Writing – original draft, Funding acquisition. CS: Investigation, Methodology, Validation, Writing – original draft. GS: Investigation, Methodology, Validation, Writing – original draft, Visualization. VC: Investigation, Writing – original draft. LC: Investigation, Writing – original draft. APa: Investigation, Writing – original draft. APo: Investigation, Methodology, Validation, Writing – review & editing. DG: Investigation, Methodology, Validation, Writing – review & editing. RC: Writing – original draft. EC: Writing – original draft, Conceptualization. CM-d-B: Conceptualization, Writing – original draft, Funding acquisition, Investigation, Methodology, Supervision, Validation, Visualization. JN-S: Conceptualization, Resources, Writing – review & editing. RS: Conceptualization, Resources, Formal analysis, Investigation, Methodology, Supervision, Validation, Visualization, Writing – original draft. FM: Conceptualization, Investigation, Methodology, Resources, Supervision, Validation, Visualization, Writing – original draft, Data curation, Writing – review & editing.

## Footnotes

^1^ https://artic.network/ncov-2019

^2^ https://www.bioinformatics.babraham.ac.uk/projects/fastqc/

^3^ https://sourceforge.net/projects/bbmap/

^4^ http://broadinstitute.github.io/picard/
