# Supplementary figures and images for: Genomic surveillance and serological profile of SARS-CoV-2 variants circulating in Macaé and nearby cities, southeastern Brazil
Source: Front Microbiol. 2024 Apr 30;15:1386271. doi: 10.3389/fmicb.2024.1386271 (PMC11091293; doi:10.3389/fmicb.2024.1386271)

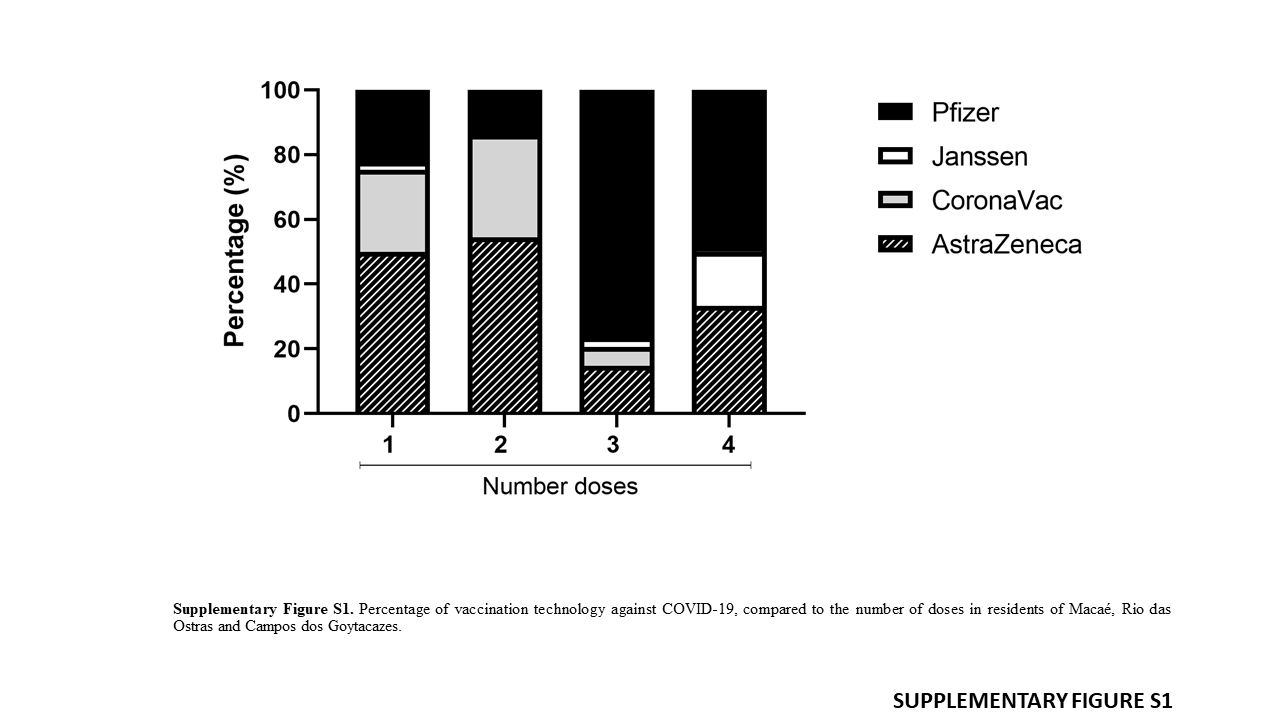

Supplement: Supplementary file 1 [file Image_1.TIF]

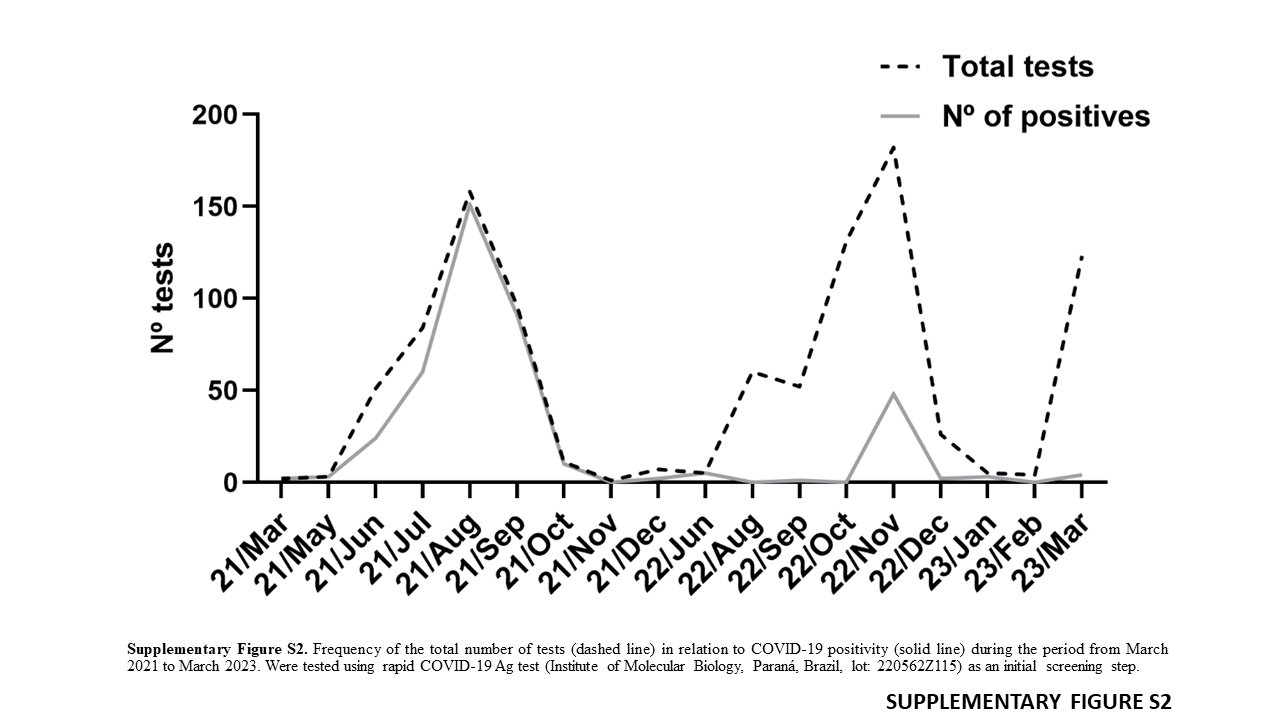

Supplement: Supplementary file 2 [file Image_2.TIF]

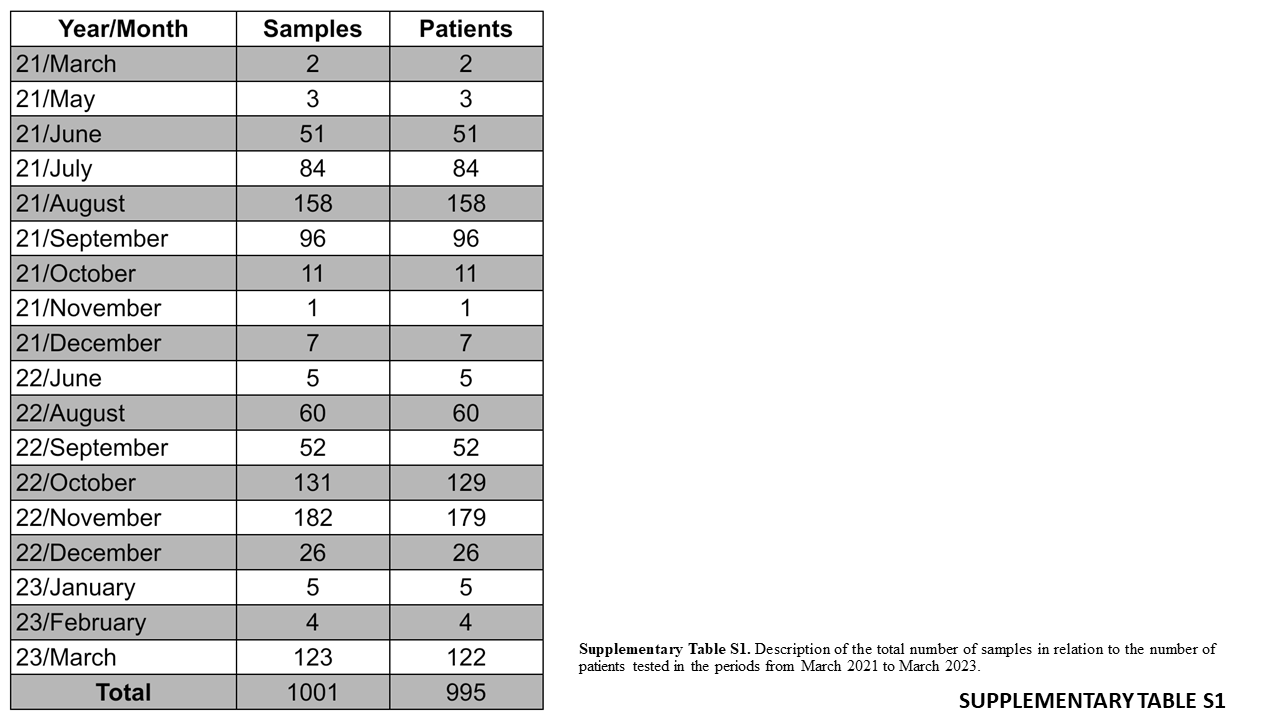

Supplement: Supplementary file 3 [file Image_3.TIF]
